# Stable cyclic (alkyl)(amino)carbene (cAAC) radicals with main group substituents

**DOI:** 10.1039/c9sc01351b

**Published:** 2019-04-08

**Authors:** Subrata Kundu, Soumen Sinhababu, Vadapalli Chandrasekhar, Herbert W. Roesky

**Affiliations:** a Universität Göttingen , Institut für Anorganische Chemie , Tammannstrasse 4 , D-37077 , Göttingen , Germany . Email: hroesky@gwdg.de; b Tata Institute of Fundamental Research Hyderabad , Hyderabad 500107 , India; c Department of Chemistry , Indian Institute of Technology Kanpur , Kanpur 208016 , India . Email: vc@iitk.ac.in

## Abstract

Isolation and characterization of stable radicals has been a long-pursued quest. While there has been some progress in this field particularly with respect to carbon, radicals involving heavier p-block elements are still considerably sparse. In this review we describe our recent successful efforts on the isolation of stable p-block element radicals particularly those involving aluminum, silicon, and phosphorus.

## Abstract 2

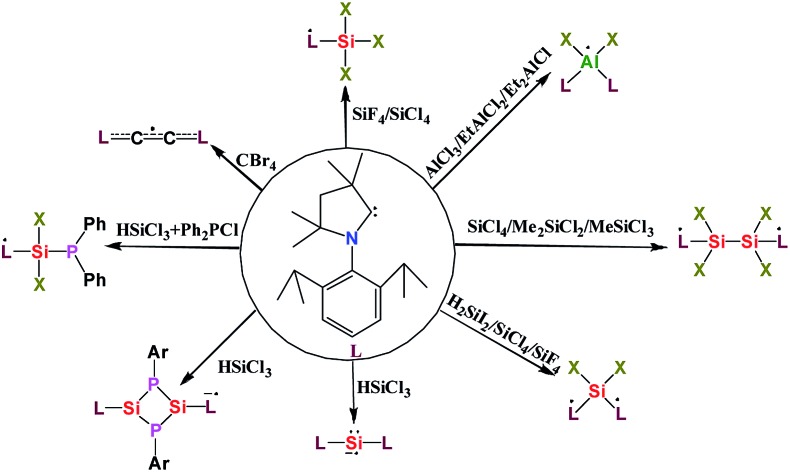
Recent attempts to isolate cyclic (alkyl)(amino)carbene stabilized radicals of p-block elements have been described here.

## Introduction

Radical chemistry has long been of interest both from academic and practical points of view.^1^ Among the most widely used radical chemistry in industrial processes is the low-density polyethylene manufacture. In addition, radical chemistry is widely utilized in organic synthesis and has implications in many biological processes including cell damage.^2^,^3^ Indeed, scavenging radicals from cells forms an important aspect of the cell machinery in biological systems.^4^ In view of this ubiquity and importance of radicals there has naturally been an interest in studying radicals both in their transient and isolated forms. While spectroscopic tools such as electron paramagnetic resonance have been of considerable utility in throwing light on the nature of the radicals, isolating the latter and characterizing them, particularly in the solid-state, has been fraught with considerable challenges in view of the reactivity of these systems. While metal-based radical systems involving coordination complexes have been far more amenable for isolation and characterization similar examples involving carbon and other main-group elements, although known, are still rare. Surprisingly, however, nature stabilizes molecular oxygen in its biradical form, quite readily. Open-shell main-group element compounds containing unpaired electrons are unstable for several reasons including the fact that many of these prefer to adopt closed-shell configurations and they readily react with any available chemical entity.^5^ Considering this propensity for reactivity, typical methods for stabilizing main group element-based radicals mainly involve delocalizing the unpaired electron on the overall molecular scaffold to achieve thermodynamic stability and/or by employing sterically encumbered groups around the main-group element such that the corresponding radicals are stabilized kinetically and are prevented from reacting with other species.^5^

Fortuitously, during this pursuit of main-group element based radicals, progress in another aspect of reactive species *viz.*, singlet carbenes is aiding the former.^6^ Thus, singlet carbenes are those that have a lone pair and a vacant orbital and have long been elusive. However, N-heterocyclic carbenes (NHCs) which are accessed by the deprotonation of an *N*,*N*′-disubstituted imidazolium (or other azolium) salt are known to be stable and have been shown to be strong σ-donors with negligible π-accepting character.^7^ Recent reports indicate that the substituents on the carbene center play a crucial role on the σ-donor ability as well as π-electron accepting properties.^8^ Bertrand *et al.* in 2005 reported that cyclic (alkyl)(amino)carbenes (cAACs) which have a nitrogen and a carbon flanking the carbene center have better σ-donor ability as well as better π-electron accepting properties than conventional NHCs.^9^ The empty p-orbital of cAACs can indeed engage in π-back bonding interactions and delocalize the electron density and at the same time stabilize an electron deficient center by σ-donation. This dual nature of the cAACs is becoming extremely promising in catalytic applications. Indeed, there have been reports on the activation of small molecules with the free carbenes themselves.^10^

**Scheme 1:**
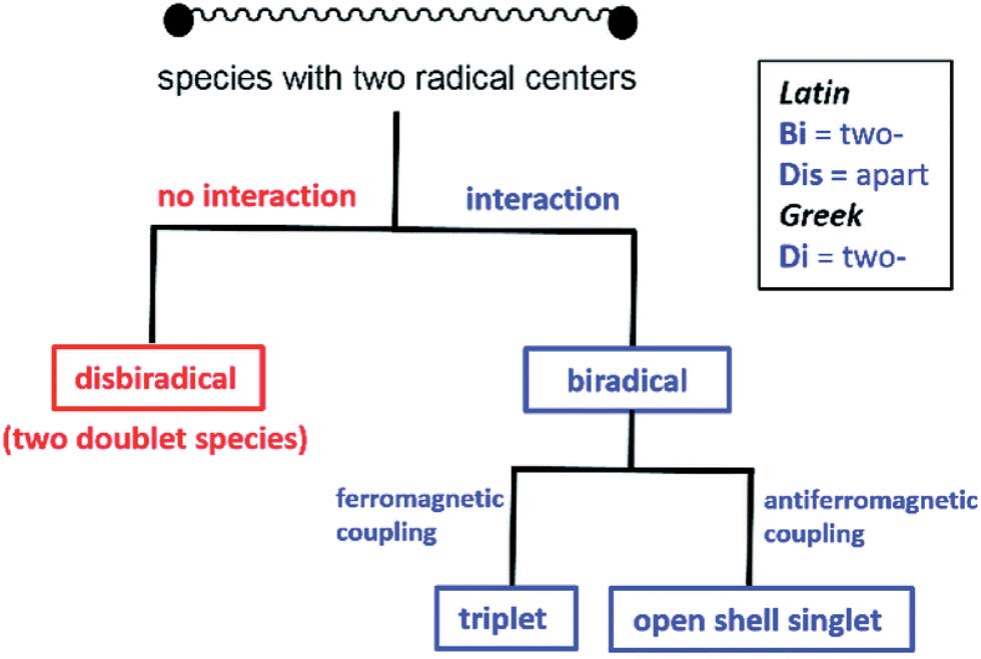
Definition for disbiradicals and biradicals used in this article (reproduced from ref. 13*a* with permission from the Royal Society of Chemistry).

In recent years, the electrophilicity of cAACs has been utilized for accessing compounds of low valent transition metals – as well as main group elements, several of them displaying paramagnetic behaviour.^11^ These cAAC-supported compounds containing low valent elements are an emerging class that have seen considerable advances in the last decade.^12^ In this review, we summarize the stable radicals of p-block elements by utilizing cAACs as ligands. There is already an elegant review from Bertrand's group on the recent developments on cAAC ligands as well as one on the isolation of stable radicals by using carbene ligands.^12^ However, this field is fast moving with many new discoveries and we feel the need to highlight the important findings in this area involving stable radicals of p-block elements. We will mainly focus results obtained from our laboratory; however, relevant research from other groups will be mentioned wherever appropriate. Few relevant examples of NHC-stabilized stable radicals of p-block elements are also mentioned. Before we begin the discussion we would like to define the notations used in this article, which will follow recent definitions outlined by Schulz (Scheme 1).13*a*

### Monoradical

When an atom or a functional group or a molecule contains an unpaired electron, it is called monoradical. An electron has a spin quantum number *S* = 1/2 so the spin multiplicity (2*S* + 1) of a monoradical is two, hence they are also called as doublet species.13*b*

### Disbiradical and biradicals (synonym diradicals)

When a molecule contains two unpaired electrons, it is termed as disbiradical or biradical.^13^ The two electrons of the molecule may interact with each other. For a disbiradical the electron exchange interaction (*J*) between the two radicals is negligible or nearly negligible (*J* ≈ 0) due to the significant distance (*r*) between them or an orthogonal orientation between them. So the disbiradicals are like two doublets within the same molecule.

A strong interaction between two radicals leads to two classical situations (a) both spins align in the same direction (↑↑; *S* = 1, 2*S* + 1 = 3; triplet) and it is called triplet biradical (b) two spins align in opposite direction to each other (↑↓; *S* = 0, 2*S* + 1 = 1; singlet) and it is called as a singlet biradical or open shell singlet (OSS). However, in both the cases the two electrons occupy two separate orbitals. A triplet biradical has a positive exchange interaction (*J*) while an open shell singlet has a negative exchange interaction. The exchange interaction between two electrons in a closed-shell singlet (CSS) molecule (non radical, both the electron occupies the same orbital) is extremely high and negative in magnitude.

### Biradicaloid (synonym diradicaloid)

Often refers to a biradical species in which the two radical centres interact significantly. In general, a relatively small energy gap between their lowest energy singlet and triplet states is observed for singlet biradicals. By increasing the HOMO–LUMO energy gap, the stability of the biradicals increases. When the LUMO occupancy of a molecule reaches zero, they are called as closed-shell molecules. However, the LUMO occupancy is not negligible for biradicaloids due to the small HOMO–LUMO energy gap and biradicaloids are relatively more reactive compared to closed-shell molecules.13*b*

The singlet biradicals do not have a net magnetic moment, so they are diamagnetic and are EPR silent. Monoradicals show one set of EPR resonance [due to allowed (Δ*m*_s_ = ±1) transition] while two sets of EPR signals are observed for a triplet biradical [due to both allowed (Δ*m*_s_ = ±1; *m*_s_ = –1 → 0 and 0 → +1) and forbidden (Δ*m*_s_ = ±2; *m*_s_ = –1 → +1) transitions]. Several theoretical methods are available to study radicals, biradicals and biradicaloids. The energy difference can also be calculated by several theoretical methods. Although, there are some limitations of different levels of theories, they are extremely helpful to study bond and spin density distribution of these species.

## Group 13 radicals

**Fig. 1:**
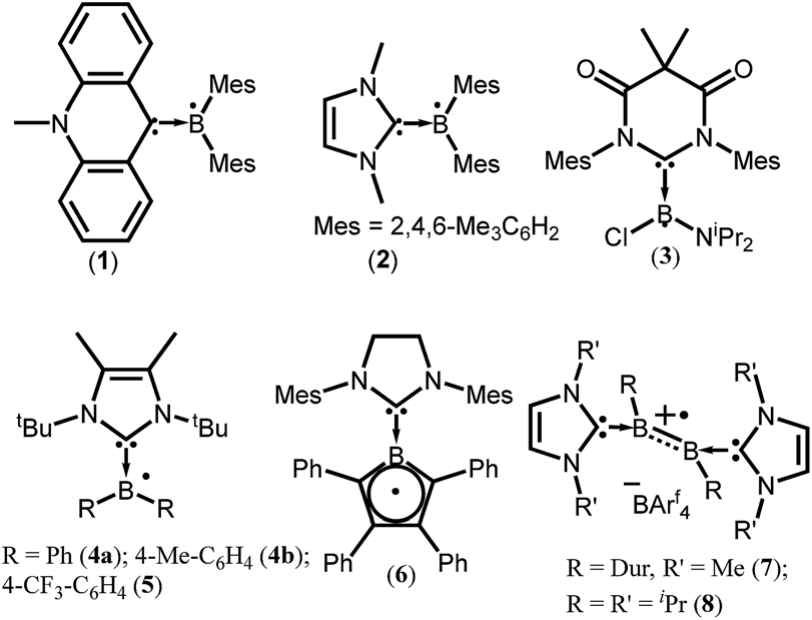
NHC ligated boryl monoradicals. The arrow indicates the donor–acceptor interaction.

To isolate group 13 element-based radicals, the strong σ-donor ability of the N-heterocyclic carbenes (NHCs) has been utilized. A review by Kinjo *et al.* summarizes all the boron containing radical species.^14^ In 2007, Gabbaï *et al.* isolated the first structurally characterized carbene-supported neutral boryl radical (Mes_2_BMe-acridine)˙ (Mes = 2,4,6-Me_3_C_6_H_2_; acridine = C_13_H_9_N) (**1**) by the reduction of the corresponding borenium cation with magnesium metal (Fig. 1).15*a* The EPR spectrum of **1** indicates that the unpaired electron is mainly delocalized over the acridinyl moiety with a small contribution on the boron atom (*a*(^11^B) = 2.55 G). After 2 years (in 2009), the same group has reported another successful synthesis of NHC-supported neutral boryl radical [(IMe)BMes_2_]˙ (IMe = 1,3-dimethylimidazol-2-ylidene) (**2**) (Fig. 1).15*b* The EPR spectrum of **2** suggested that the delocalization of the spin density over the boron center, mesityl groups, and the entire carbene framework (*a*(^11^B) = 7.90 G). Subsequently, a few more NHC-stabilized boryl radicals have been isolated; some examples (**3–8**) are listed in Fig. 1.^16^ The EPR spectrum of **6** shows a four-line signal arising from couplings with the two boron isotopes [*a*(^11^B) = 3.02 G; *a*(^10^B) = 1.02 G], which indicate the delocalization of the unpaired electron over the borolyl ring. The EPR spectra of **7–8** indicate that the spin density is mainly concentrated on the boron–boron bond. It has been observed that the spin density of most of the NHC stabilized boryl radicals is delocalized over the substituents attached to the boron atom.16*c* The spin density of all the group 13 species discussed here are given in Table 1.

**Table 1:** Group 13 radicals and the distribution of spin density^*a*^

Compounds	Spin density	Type of radical	Ref.
**1**	Not given	Monoradical	15*a*
**2**	Not given	Monoradical	15*b*
**3**	90% C_carbene_, 7.8% B	Monoradical	16*a*
**4a**	87% BR_2_, 13% NHC, 41% B, 3% C_carbene_	Monoradical	16*b*
**4b**	86% BR_2_, 14% NHC, 41% B, 4% C_carbene_	Monoradical	16*b*
**5**	90% BR_2_, 10% NHC, 39% B, 2% C_carbene_	Monoradical	16*b*
**6**	47% B, 7% C_carbene_	Monoradical	16*c*
**7**	Not given	Monoradical cation	16*d*
**8**	Not given	Monoradical cation	16*e*
**9**	49.6% C_carbene_, 27.7% B, 24.1% N_carbene_	Monoradical	17*a*
**10**	Not given	Monoradical	17*a*
**11**	Not given	Monoradical	17*b*
**12**	57% C_carbene_, 21% N_carbene_	Triplet	17*c*
**13**	Not given	Monoradical	17*d*
**14**	Mainly delocalized over the B–C_cAAC_–N_cAAC_ linkage	Monoradical	17*e*
**15**	Not given	Triplet	18*a*
**16**	27% B, 49% C_cAAC_, 24% N_cAAC_	Triplet	18*b*
**17**	50% B, 33% N_cAAC_ (total)	Monoradical cation	18*c*
**18**	49% B	Monoradical cation	18*d*
**19**	Not given	Monoradical cation	17*d*
**20**	2% Al, 29% C_cAAC_, 50% C_cAAC_, 11% N_cAAC_, 6% N_cAAC_	Monoradical	19
**21**	5.7% Al, 15% C_cAAC_, 62% C_cAAC_, 10% N_cAAC_, 2% N_cAAC_	Monoradical	20
**22**	5.1% Al, 15% C_cAAC_, 65% C_cAAC_, 9% N_cAAC_, 2% N_cAAC_	Monoradical	20

^*a*^Unless other wise mentioned all the radicals are neutral.

**Fig. 2:**
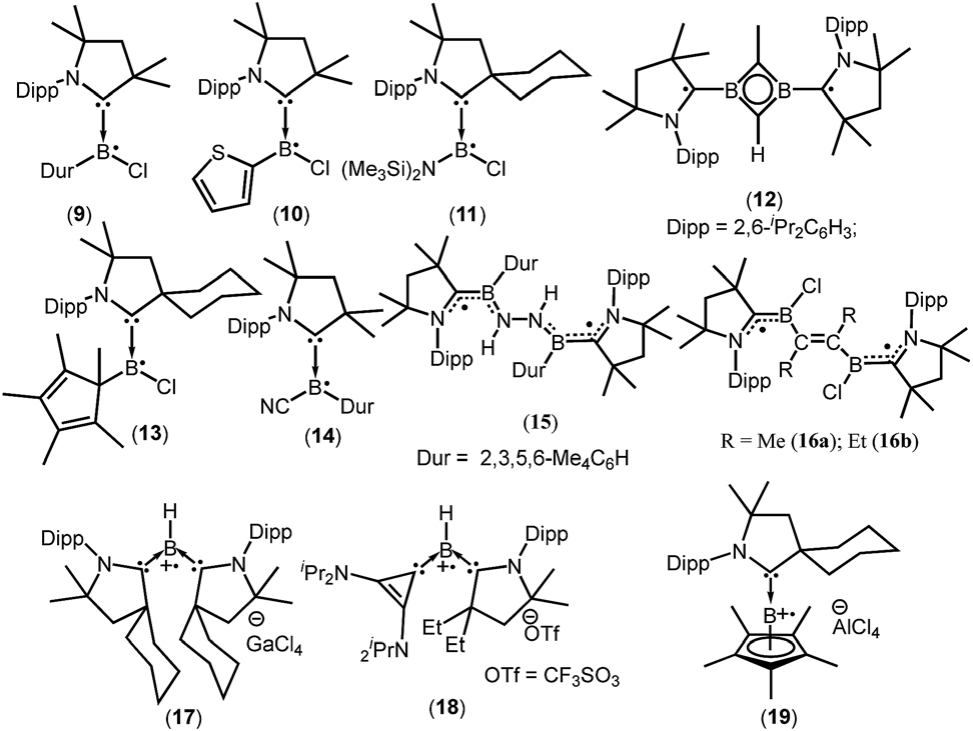
cAAC ligated boryl mono- and biradicals.

cAACs could be employed to isolate base-stabilized boryl radical with larger spin density on the boron atom and a large number of stable boryl radicals have been isolated and structurally characterized (**9–19**; Fig. 2).^14^,^17^,^18^

The success of cAACs can be attributed to their stronger σ-donating and π-accepting properties, in comparison to NHCs, and also cAACs are sterically more demanding owing to the presence of a quaternary carbon atom adjacent to the carbene center.

In 2014, Braunschweig *et al.*, Bertrand *et al.* and Stephan *et al.* independently reported the isolation and structural characterization of cAAC-supported neutral boryl radicals (^*Me2*^cAACBClDur)˙ (**9**),17*a* (^*Me2*^cAACBCl(C_4_H_3_S))˙ (**10**),17*a* and (^*cy*^cAACBClN(SiMe_3_)_2_)˙ (**11**),17*b* respectively (Fig. 2). The EPR signal of **9** was detected as a 1 : 1 : 1 triplet due to the hyperfine interaction with the ^14^N nucleus (*a*(^14^N) = 19 MHz) and the weaker hyperfine couplings, including coupling to boron were not resolved due to the spin delocalization onto the cAAC unit. However, attempts to determine the solid state structure of **10** failed. On the other hand, a broad signal in the EPR spectrum of **10** was observed, clearly confirming the presence of the expected radical species.17*a* Aminochloroboryl radical **11** exhibits similar hyperfine coupling constants in the EPR spectrum [*a*(^11^B) = 4.7, *a*(^14^N) = 18.4 and *a*(^35^Cl) = 2.5 MHz] compared to the arylchloroboryl radical (**9**). Braunschweig *et al.* isolated and crystallographically characterized a four membered 2π-aromatic system (**12**; Fig. 2) from the reaction of bis(^*Me2*^cAAC)B_2_ with propyne. Compound **12** has two unpaired electrons which are localized on the cAACs.17*c* The EPR spectrum of a frozen-solution sample of **12** in 2-methyltetrahydrofuran shows a weak half-field signal and a four-line spectrum in the *g* = 2 region which can be attributed to the triplet state of **12**. In 2016, Chiu *et al.* reported the persistent boron-containing radical (^*cy*^cAACBClCp*)˙ (Cp* = C_5_Me_5_) (**13**) (Fig. 2).17*d* The EPR signal of **13** at *g* = 2.0047 can be simulated with the hyperfine couplings to nitrogen (*a*(^14^N) = 6.50 G), boron (*a*(^11^B) = 1.60 G, *a*(^10^B) = 0.54 G), and chlorine (*a*(^35^Cl) = 1.50 G, *a*(^37^Cl) = 1.25 G) nuclei. The relatively small *a*(^11^B) coupling constant suggests that **13** should be better represented as a carbon-centered radical. The same group has reported a cyanide-containing boron radical (^*Me2*^cAACBCNDur)˙ (**14**; Fig. 2) which was characterized by EPR spectroscopy as well as X-ray diffraction.17*e* EPR spectroscopy of **14** revealed a multiple-line spectrum with *g*_iso_ = 2.0029, indicative of hyperfine interactions with both the nitrogen and boron atoms. The observed hyperfine coupling constants [*a*(^14^N) = 18.3 MHz and *a*(^11^B) = 10.7 MHz] suggest that the unpaired electron is primarily delocalized over the B–C_cAAC_–N_cAAC_ linkage. Recently a dipotassium complex {[(^*Me2*^cAAC)DurB]_2_(μ^2^-N_2_K_2_)} was synthesized by Braunschweig *et al.* by the *over-reduction* of [(^*Me2*^cAAC)BClDur] with an excess of KC_8_ under nitrogen atmosphere.18*a* Treatment of the dipotassium complex with distilled water led to formation of the paramagnetic compound {[(^*Me2*^cAAC)DurB]_2_(μ_2_-N_2_H_2_)} (**15**) (Fig. 2) which was characterized by single crystal X-ray diffraction and EPR spectroscopy.18*a* The solid state EPR spectrum of **15** at 290 K revealed the typical signature of a triplet state, with a half-field signal at about *g* = 4 and zero-field splitting parameters of |*D*/*hc*| = 0.021 cm^–1^ and |*E*/*hc*| = 0.00083 cm^–1^. Very recently the neutral two-carbon bridged boron-based biradicals (**16a–b**) were reported by Braunschweig *et al.*18*b* In frozen toluene solution, **16a–b** show evidence for a triplet state with a weak half-field signal. Theoretical calculations (using meta-NGA functional MN12Lin conjugation with the 6-311G(d,p) Pople basis set) indicate that singlet–triplet gap for **16a–b** is nearly zero and each free electron is effectively delocalised on both sides of the molecule in the N_cAAC_–C_cAAC_–B π system; the calculated spin densities are (B 0.27; C_cAAC_ 0.49; N_cAAC_ 0.24).18*b*

In contrast to anionic and neutral boron radicals, cationic boron radicals are extremely rare due to the intrinsic electron deficient nature of boron. However, the strong electron donating property of cAACs has been exploited to isolate cationic boron radicals. In 2011 Bertrand *et al.* first reported a bis(cAAC)borylene adduct [(^*cy*^cAAC)_2_BH]. The boron atom of [(^*cy*^cAAC)_2_BH] is in a formal +1 oxidation state with an active lone pair on boron and it behaves as an electron-donor.18*c* One-electron oxidation of (^*cy*^cAAC)_2_BH by the use of gallium trichloride, quantitatively afforded the first crystallographically characterized radical cation **17** [(^*cy*^cAAC)_2_BH]˙^+^[GaCl_4_]^–^ (Fig. 2).18*c* The EPR spectrum (*g* = 2.0026) of **17** displays couplings with boron (*a*(^11^B) = 6.432 G), hydrogen (*a*(^1^H) = 11.447 G), and two nitrogen nuclei (*a*(^14^N) = 4.470 G). The EPR data and theoretical calculations indicate that the spin density is primarily located on the boron atom (50%) and the nitrogen atoms of the carbenes (33% total). The same research group in 2014 reported a bis-carbene stabilized radical cation **18** [(^*Et*^cAAC)(L2)BH] ˙^+^[CF_3_SO_3_]^–^ (L2 = cyclopropenylidene) (Fig. 2) whose radical nature is persistent for several hours at room temperature.18*d* In contrast to **17,** the cationic radical **18** was prepared by a single electron reduction of the bis(carbene) boronium triflate salt. The EPR spectrum of **18** displays couplings with hydrogen (*a*(^1^H) = 10.065 G), boron (*a*(^11^B) = 4.994 G), and one nitrogen (*a*(^14^N) = 6.627 G) which indicates that the unpaired electron is mainly delocalized over the cAAC and BH fragments, with little distribution on the cyclopropenylidene ligand. DFT calculations confirm that the spin density distribution is consistent with the EPR observations. Interestingly, in 2016, the Chiu group reported that treatment of the cAAC-stabilized boron cation [Cp*B(^*cy*^cAAC)]^2+^2[AlCl_4_]^–^ with a half equivalent of tetrakis(dimethylamino)ethylene generated the boron-containing radical cation **19**.17*d* The EPR spectrum (*g* = 2.0032) can be simulated with hyperfine coupling constants of (*a*(^11^B) = 9.00 G), (*a*(^10^B) = 3.00 G), and (*a*(^14^N) = 6.20 G).17*d*

**Fig. 3:**
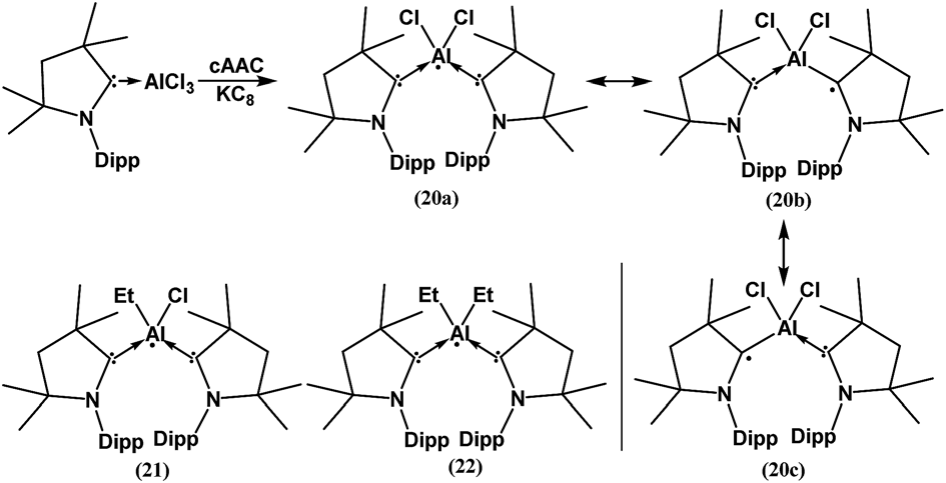
cAAC-supported neutral aluminium monoradicals (**20–22**). As an example, a general synthesis and possible resonance structures of **20** are given.

**Fig. 4:**
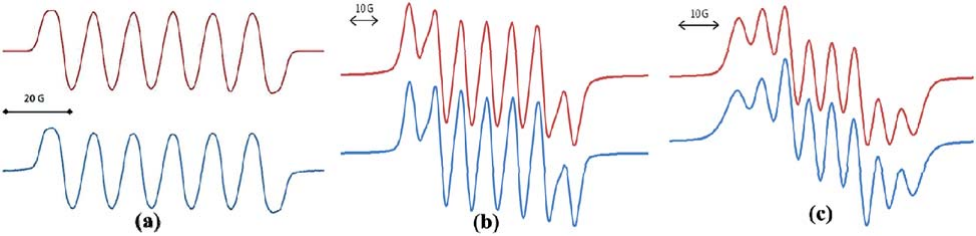
Room-temperature (a) EPR spectrum of **20** simulation with (*g* = 2.0055), *a*(^27^Al) = 12.5 G; *a*(2 ^35,37^Cl) = 1.9 G; *a*(2 ^14^N) = 0.25 G; (b) EPR spectrum of compound **21** simulation with *a*(^14^N) = 4.3 G, *a*(^27^Al) = 8.3 G; (c) EPR spectrum of compound **22** at room temperature simulation with *a*(^14^N) = 4.95 G, *a*(^27^Al) = 5.2 G (Top: simulated spectrum and Bottom: experimental spectrum).

In spite of the progress on boron-centered radicals, as discussed above, there has been no report on an aluminum-centered radical till very recently. In 2017, we first reported the neutral radical of aluminum ((^*Me2*^cAAC)_2_AlCl_2_)˙ (**20**; Fig. 3), which was synthesized by the reduction of the ^*Me2*^cAAC → AlCl_3_ adduct with KC_8_ in the presence of another equivalent of cAAC (Fig. 3).^19^ The EPR spectrum is dominated by a sextet that results from coupling of the unpaired electron with one aluminum nucleus (^27^Al, *I* = 5/2) (Fig. 4a). The hyperfine splitting [*a*(^27^Al) = 12.5 G] indicates a metal-based spin. However, the unusual aluminum radical **20** can be equally well described by the three resonance forms that are shown in Fig. 3 according to the quantum-chemical calculations at the BP86/TZVPP level of theory. Following this result we also reported the analogous cAAC-stabilized mono- and diorgano aluminum radicals [((^*Me2*^cAAC)_2_AlClEt)˙ (**21**) and ((^*Me2*^cAAC)_2_AlEt_2_)˙ (**22**)] (Fig. 3).^20^ The observed hyperfine splitting for **21** is *a*(^14^N) = 4.3 G and *a*(^27^Al) = 8.3 G while for **22** it is *a*(^14^N) = 4.95 G and *a*(^27^Al) = 5.2 G (Fig. 3b and c). Coupling is observed from *only one* nitrogen atom in both the cases confirming the localization of spin at a single cAAC ligand which is also observed from the asymmetric C_carbene_–Al bond distances [2.1507(13) and 1.9913(13) Å] found in the molecular structure of **21**. Thus, with the replacement of chloride substituents by alkyl groups the spin density changes from aluminum to the cAAC carbon. The sequence of the hyperfine coupling constants was reproduced by DFT calculations.

## Group 14 radicals

**Fig. 5:**
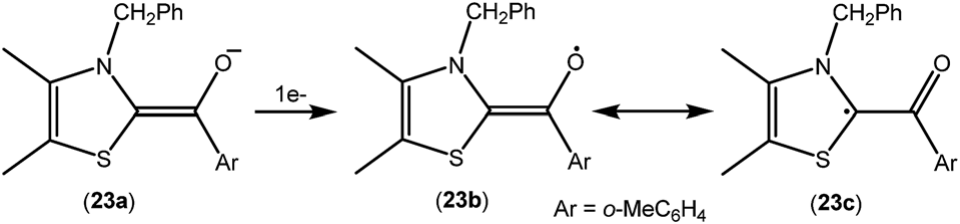
Oxidation of the thiazolylidene based enolate to a carbon-radical.

**Fig. 6:**
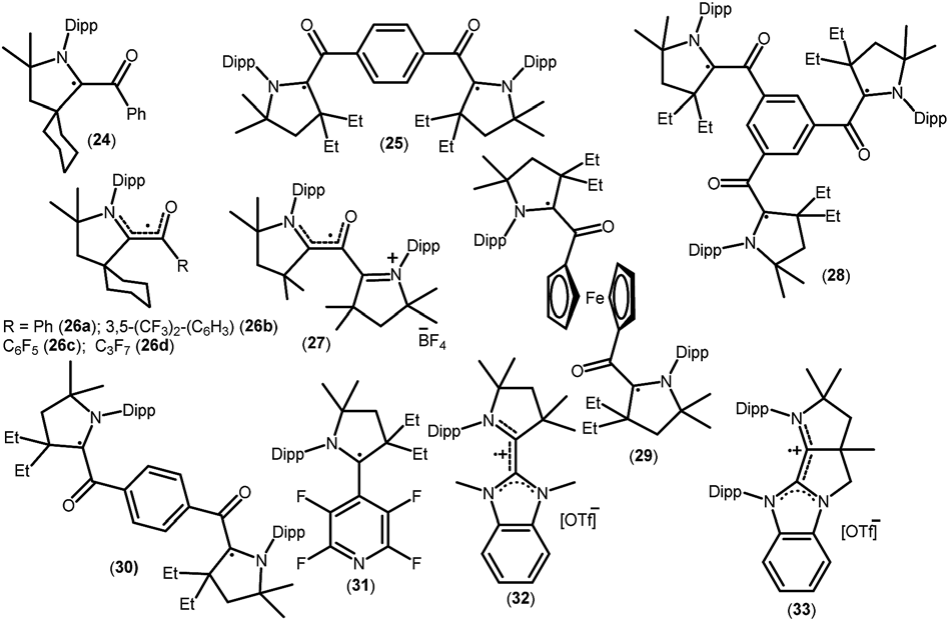
cAAC-derived carbon radicals.

**Fig. 7:**
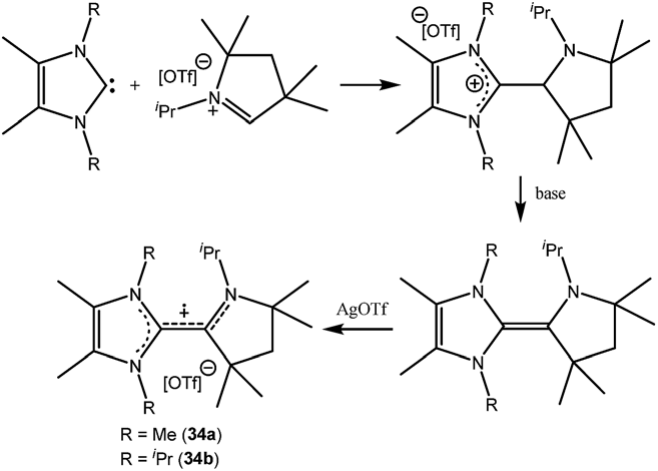
Monoradical cations derived from cAACs.

**Fig. 8:**
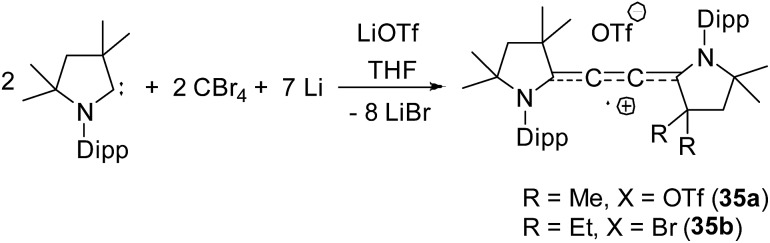
Synthesis of di-cAAC stabilized C_2_˙^+^.

As mentioned above, cAACs are efficient in the stabilization of paramagnetic species. In this section, persistent and stable group 14 radicals and radical ions associated with cAACs will be discussed. Table 2 summarizes the spin densities of group 14 radical species. Fukuzumi *et al.* reported the first carbene-stabilized carbon-based radical (Fig. 5) in 1997 which is persistent for several hours at room temperature. As shown in Fig. 5 the carbon-based radicals **23b–c** were generated by the electrochemical oxidation of the corresponding thiazolylidene enolate.^21^ After this, several carbene-stabilized carbon radicals have been reported. Literature data indicate that most of the persistent and stable radicals of carbon contain cAACs because of the delocalization of the spin density over the ligand (**24–33**) (Fig. 6).^12^,^22^ In continuation of the theme on isolation of stable carbon centred radicals our group has contributed by isolating two types of cationic radicals (**34–35**; Fig. 7 and 8).

**Table 2:** Group 14 element containing radicals and the distribution of spin density

Compounds	Spin density	Type of radical	Ref.
**23**	Not given	Monoradical	21
**24**	40% C_cAAC_, 28% O, 24% N_cAAC_, 6% C	Monoradical	22*a*
**25**	Not given	Disbiradical	22*a*
**26a**	42% C_cAAC_, 28% O	Monoradical	22*b*
**26b**	39% C_cAAC_, 29% O	Monoradical	22*b*
**26c**	37% C_cAAC_, 31% O	Monoradical	22*b*
**26d**	31% C_cAAC_, 33% O	Monoradical	22*b*
**27**	19% C_cAAC_, 36% O	Monoradical	22*b*
**28**	Not given	Dis-triradical	22*a*
**29**	Not given	Triplet	22*c*
**30**	Not given	Triplet	22*c*
**31**	41.1% C_cAAC_, 21.1% N_cAAC_, 25% (C_4_F_4_N)	Monoradical	22*d*
**32**	33% C_cAAC_, 23% N_cAAC_, 10% C_NHC_	Monoradical cation	22*e*
**33**	25% C_cAAC_, 20% N_cAAC_, 9% C_NHC_	Monoradical cation	22*e*
**34**	Not given	Monoradical cation	23
**35a**	60% on C_4_ unit, 40% N_cAAC_	Monoradical cation	24
**35b**	44% N_cAAC_, 24% C_cAAC_, 31% C_cAAC_	Monoradical cation	25
**36c**	43% C_cAAC_, 15% N_cAAC_, 39% C_^*t*^Bu_, 2.5% C_ment_	Monoradical	26*b*
**36d**	45% C_cAAC_, 17% N_cAAC_, 38% C_CPh3_	Monoradical	26*b*
**37a**	Not given	Biradicaloid	26*c*
**37b–c**	Not given	Disbiradical	26*c*
**38a**	58% cAAC, 38% linker	Monoradical cation	26*d*
**38b**	50% cAAC, 50% linker	Monoradical cation	26*d*
**38c**	52% cAAC, 48% linker	Monoradical cation	26*d*
**39**	(37–45%) C_carbene_, (8–11%) N_carbene_, (19–23%) C_*ortho*_, (19–29%) C_*para*_	Monoradical	26*e*
**40**	Not given	Biradicaloid	26*f* and *g*
**41**	Not given	Disbiradical	27
**42**	Not given	Disbiradical and OSS	28
**43**	90% C_cAAC_, 10% N_cAAC_	Disbiradical	29
**44**	65% C_cAAC_, 18% N_cAAC_	OSS	30
**45a–b**	71% C_cAAC_, 22% N_cAAC_, 6% Si	Monoradical	31
**45c**	66% C_cAAC_, 25% N_cAAC_	Monoradical	30
**46**	80% C_cAAC_, 16% N_cAAC_, 5% Si	Monoradical	32
**47**	Not given	CSS biradicaloid	33
**48**	Not given	Monoradical anion	35
**49**	Not given	OSS	36
**50**	95% C_cAAC_, 5% N_cAAC_	Disbiradical	29
**51**	92% C_cAAC_, 8% N_cAAC_	Disbiradical	29
**52**	52% Si	Monoradical cation	37

Organic cationic radicals [^*Me2*^cAAC-NHC]˙^+^ OTf^–^ (**34**, Fig. 7) were obtained from the one-electron oxidation of the NHC-cAAC heterodimers which were synthesized by the reaction of NHC with a cyclic iminium salt, followed by deprotonation.^23^ X-band EPR measurements of the radical cations (**34**) in THF at room temperature revealed that the unpaired electron couples with all the three nitrogen atoms as indicated by the presence of hyperfine coupling [**34a**: 16.8, 13.2, and 12.0 MHz; **34b**: 19.8, 12.7, and 10.4 MHz]. We and Bertrand and co-workers independently reported the radical cations [(^*Me2*^cAAC)_2_C_2_]˙^+^ OTf^–^ (**35a**) and [(^*Et2*^cAAC)_2_C_2_]˙^+^Br^–^ (**35b**) respectively, which can be viewed as a C_2_˙^+^ fragment, stabilized by two carbenes (Fig. 8).^24^,^25^ The synthetic routes used to prepare these di-carbene-stabilized C_2_˙^+^ compounds were, however, different. We obtained the dark red coloured cationic radical species [(^*Me2*^cAAC)_2_C_2_]˙^+^OTf^–^ (**35a**) from the reduction of CBr_4_ with 3.5 equivalents of lithium sand in the presence of one equivalent of ^*Me2*^cAAC·LiOTf. On the other hand, Bertrand and co-workers isolated [(^*Et2*^cAAC)_2_C_2_]˙^+^Br^–^ (**35b**) from the reaction of [(^*Et2*^cAAC)Br]^+^Br^–^ with LiC_2_SiMe_3_. Since the radical cations **35a** and **35b** are readily prepared and are surprisingly air stable, these results open up the possibility for the preparation of a variety of organic mixed valence systems using cAACs as stabilizing groups along with different radical cation spacers. The relatively small ^14^N hyperfine coupling [*a*(^14^N) = 5.3 G; quintet 1 : 2 : 3 : 2 : 1 for two equivalent nitrogen atoms] indicates the concentration of spin on the C_4_ backbone with limited participation of the nitrogen center of the carbenes.

**Fig. 9:**
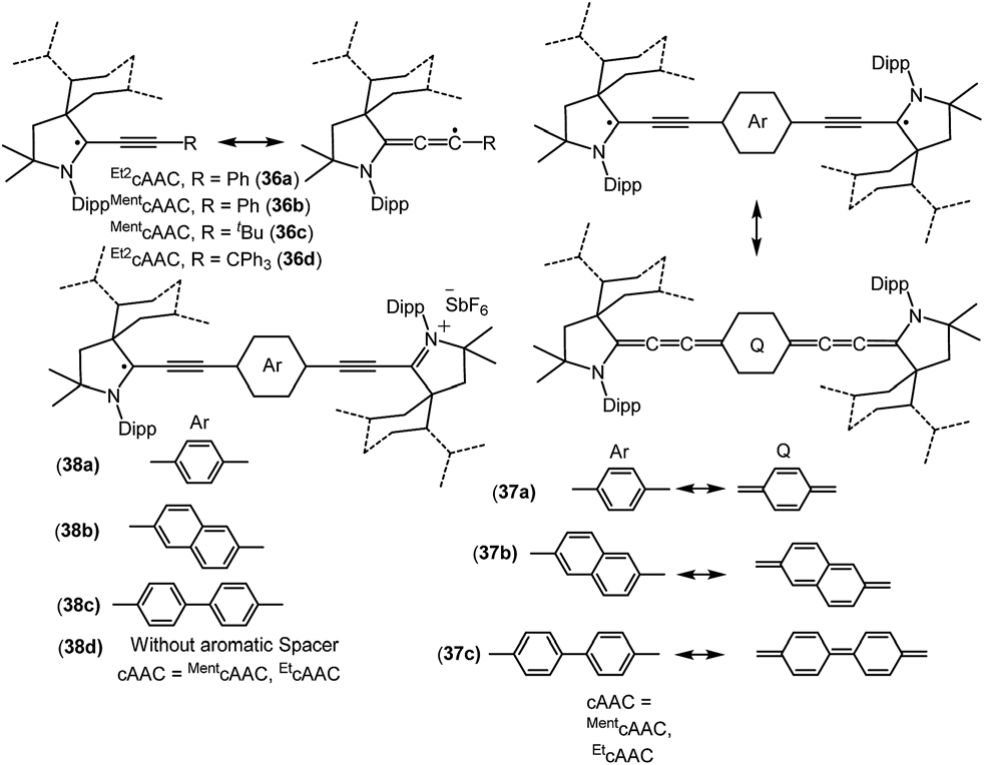
cAAC stabilized mono-, di- and biradicals over extended carbon systems.

Bertrand and co-workers have reported the synthesis of the allenyl/propargyl radicals {[^*Et2*^cAAC(C_2_Ph)]˙ (**36a**); [^*Ment*^cAAC(C_2_Ph)]˙ (**36b**); [^*Ment*^cAAC(C_2_^*t*^Bu)]˙ (**36c**); [^*Et2*^cAAC(C_2_CPh_3_)]˙ (**36d**); Fig. 9}, by the one-electron reduction of the corresponding alkynyl-iminium salts [(**36^+^a–d**)(SbF_6_^–^)] using cobaltocene.26a,b The desired alkynyl-iminium precursors [(**36^+^a–d**)(SbF_6_^–^)] were prepared in two steps. In the first step, oxidative insertion of the sp-hybridized C–H bonds of the alkyne to the cAAC centre occurred at room temperature. Subsequently, hydride abstraction from the oxidatively inserted product by a stoichiometric amount of DDQ, followed by treatment with NOSbF_6_, afforded alkynyl-iminium salts in good yield. The stability of these radicals vastly differ, being short-lived, [^*Et2*^cAAC(C_2_Ph)]˙ (**36a**), or stable, [^*Et2*^cAAC(C_2_CPh_3_)]˙ (**36d**), depending on the nature of the cAAC and the alkyne substituents. Single crystal X-ray diffraction studies indicate that **36a–b** dimerize in the solid state; however, **36d** exists as a monomer in the solid state. The EPR spectrum of **36b** shows the hyperfine couplings with N and the *ortho*, *meta* and *para* Hs and with one H atom of the menthyl group [*a*(^14^N) = 4.3 G; *a*(^1^H) = 2.9 G; *a*(^1^H) = 0.5 G; *a*(^1^H) = 2.8 G; *a*(^1^H) = 2.3 G]. The X-band EPR spectrum of **36d** in solution reveals a triplet (*a*(^14^N) = 4.45 G).26*b*

**Fig. 10:**
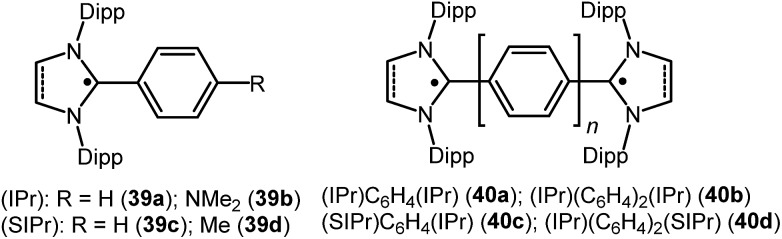
NHC stabilized radicals over extended carbon systems.

Similar to the above, an interesting study on cAAC supported compounds, [1,4-[(cAAC)˙C_2_]_2_(Ar) where Ar = C_6_H_4_ (**37a**); C_10_H_6_ = (**37b**); C_12_H_10_ (**37c**)] were recently reported by Bertrand *et al.* (Fig. 9).26*c* Analogous to the synthesis of **36**, bis(2-acetylenyliminium) salts which were obtained from the reactions of cAACs and bis(acetylenes) in the initial step, undergo reduction with 2 equivalents of cobaltocene to give the respective biradicaloid/disbiradical compounds **37a–c** (Fig. 9).26*c* The (U)CAM-B3LYP/6-31G** and (U)M05-2X/6-31G** calculations indicate that **37a–c** possess biradical and quinoid resonance structures as indicated in Fig. 9 and have a OSS ground state with varying degrees of biradical character in combination with small singlet–triplet gaps. It has been observed that upon increasing the length of the spacer, the properties of the compounds approach those of monoradicals. The EPR spectrum of 1,4-[(^*Et2*^cAAC)˙C_2_]_2_ C_6_H_4_ (**37a**) in pentane at room temperature shows a triplet with isotropic hyperfine coupling constants with nitrogen (*a*(^14^N) = 5.6 G). In the solid state, at elevated temperature (325 K), a half-field signal (*g* = 1682 G) was detected, demonstrating the triplet nature of **37a**. Theoretical calculations ((U)CAM-B3LYP/6-31G** and (U)M05-2X/6-31G**) reveal a singlet ground state with considerable triplet character, *i.e.*, population of the antibonding lowest unoccupied molecular orbital (LUMO) by 0.3 electrons (biradical index of 0.3), and a singlet–triplet gap of 15.2 kcal mol^–1^ were obtained for **37a**. With the increased spacer length the radical did not interact, so **37b–c** are disbiradicals. Bertrand and co-workers have also reported the related radical-cations **38a–c** [(^*Ment*^cAAC)˙C_2_(Ar)C_2_^*Ment*^cAAC)(SbF_6_) where Ar = C_6_H_4_ (**38a**); C_10_H_6_ (**38b**); C_12_H_10_ (**38c**) (Fig. 9)] featuring aromatic spacers and acetylenic units, as well as without an aromatic spacer (^*Et2*^cAAC)˙C_4_(^*Et2*^cAAC)(SbF_6_) (**38d**) (Fig. 9) which have been synthesized from the corresponding bis(2-acetylenyliminium) salts by using 1 equiv. of cobaltocene or zinc as a reducing agent.26*d* Compounds **38a–d** are EPR active and show characteristics of mixed valent compounds. The X-band EPR spectrum of **38d** in THF at room temperature can be simulated by involving coupling with two equal nitrogen nuclei, *a*(^14^N) = 4.14 G. Interestingly, the EPR spectrum of [(^Et^cAAC)_2_C_2_]^+^˙(**35b**) shows similar hyperfine splitting pattern but with larger N-coupling constants (*a*(^14^N) = 5.3 G), which indicate a decreased spin density on the nitrogen atom of cAAC in (^*Et2*^cAAC)˙C_4_(^*Et2*^cAAC)(SbF_6_) (**38d**) and thus a larger electron delocalization. Similarly, the hyperfine coupling constants decrease with the increase of the spacer length [**38a** (*a*(^14^N) = 2.70 G), **38b** (*a*(^14^N) = 2.30 G), and **38c** (*a*(^14^N) = 2.22 G] indicating a symmetrical delocalization of the unpaired electron over the central spacer.26*d* Very recently classical NHCs have been employed by Ghadwal *et al.* to isolate analogous carbon based radicals (**39–40**; Fig. 10) where the spin density is delocalized over an extended π system.26e–g

**Fig. 11:**
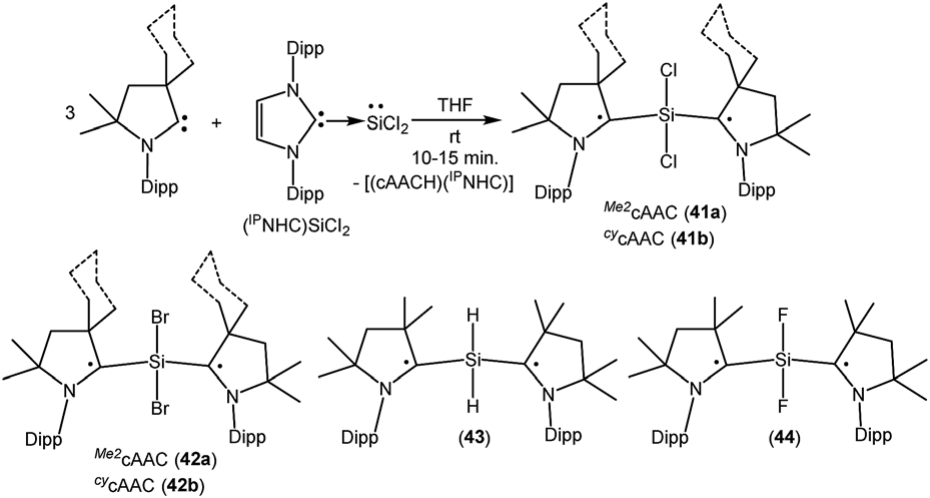
cAAC coordinated SiR_2_ (R = Cl, Br, H, F) dis- and biradicals.

As with carbon-centered radicals, the strong σ-donor and π-acceptor properties of cAACs have been exploited to isolate various neutral and ionic radicals of silicon. The most striking examples of the ability of cAACs to stabilize neutral radical species of silicon have been reported by us. Thus, we have reported that the reaction of three equivalents of cAAC with one equivalent of (NHC)SiCl_2_ adduct gives deep blue coloured biradicals **41a–b** [(^*Me2*^cAAC˙)_2_SiCl_2_ (**41a**) and (^*Cy*^cAAC˙)_2_SiCl_2_ (**41b**); Fig. 11].^27^

Unlike (NHC)SiCl_2_, in compounds **41a–b** the two nonbonding electrons are not localized on silicon, rather, an unpaired electron resides on each carbene center. Moreover, we were able to isolate both the disbiradical (polymorph I) and open shell singlet (polymorph II) as two polymorphs *via* fractional crystallization of **41a**, the singlet being air stable for up to a week in the solid state. The EPR spectrum of the diluted C_6_D_6_ solution of the polymorph I of **41a** shows six hyperfine lines at room temperature (*a*(^14^N) = 5.7 G). The EPR spectrum could be simulated as a disbiradical species (two *S*_eff_ = 1/2), each electron interacting with the closest ^14^N nucleus. As expected for a disbiradical, the half field (Δ*m*_s_ = ±2 forbidden) transition was not observed either in the solid state or in solution of polymorph I of **41a**. Theoretical calculations on **41a** at the UM05-2X/SVP level using Gaussian 09 revealed that the open shell singlet state is 2.6 kcal mol^–1^ lower in energy than the triplet (3.2 kcal mol^–1^ in single-point energy calculations using the TZVPP basis set). By following a similar synthetic strategy used for **41a–b**, we also reported the bromide analogues **42a–b** [(^*Me2*^cAAC˙)_2_SiBr_2_ (**42a**) and (^*cy*^cAAC˙)_2_SiBr_2_ (**42b**)] (Fig. 11) using (NHC)SiBr_2_ as a precursor.^28^ It has been observed that the bromide analogues (**42a–b**) are comparatively less stable than the chloride analogues (**41a–b**) and are more prone to decomposition in the solution. No EPR characterization was possible for (**42a–b**); however, compound (^*cy*^cAAC˙)_2_SiBr_2_ (**42b**) was characterized by a single crystal X-ray diffraction study.

As there were no suitable precursors available for the synthesis of [(^*Me2*^cAAC˙)_2_SiH_2_] (**43**, Fig. 11), we prepared it from a direct reduction of H_2_SiI_2_ with two equivalents of KC_8_, in the presence of two equivalents of cAAC by developing a one step synthetic strategy.^29^ However unlike **41**, polymorph formation was not observed in **43** and the theoretical calculations using density functional theory (DFT) at the M06-2X/def2-TZVPP level using M06-2X/def2-SVP optimized geometries of the two molecules in the electronic singlet and triplet states suggest that the triplet state of **43** is lower in energy than the respective open shell singlet state by 9.3 kcal mol^–1^. The EPR spectrum of **43** exhibits hyperfine splitting (*a*(^14^N) = 6.2 G) and a satellite coupling (*a*(^29^Si) = 23 G). The small ^14^N hyperfine splitting is in agreement with calculations that the bulk of spin density resides on the cAAC carbon. Very recently, we have isolated the elusive SiF_2_ species as a cAAC coordinated biradical [(^*Me2*^cAAC˙)_2_SiF_2_ (**44**); Fig. 11].^30^ Compound **44** was prepared from the reduction of (^*Me2*^cAAC)SiF_4_ adduct by using two equivalents of KC_8_ in the presence of one equivalent of cAAC. Theoretical calculations on **44** at M05-2X/def2-TZVPP//M05-2X/def2-SVP level suggest that the open-shell singlet is lower in energy than the triplet and closed-shell singlet by 4.9 kcal mol^–1^ and 10.2 kcal mol^–1^ respectively. Previous calculations on (^*Me2*^cAAC˙)_2_SiCl_2_ (**41a**) also indicated that the open-shell singlet is lower in energy by 3.2 kcal mol^–1^ than the triplet. Although both forms were experimentally found as a paramagnetic polymorph I (minor component) and a diamagnetic polymorph II (major component) for **41a**, the fluoride homologue **44** appears only as a EPR-silent compound. A triplet at –29.73 ppm and a broad singlet –123.47 ppm were observed in the ^29^Si{^1^H} and ^19^F{^1^H} NMR spectra respectively at low temperatures.

**Fig. 12:**
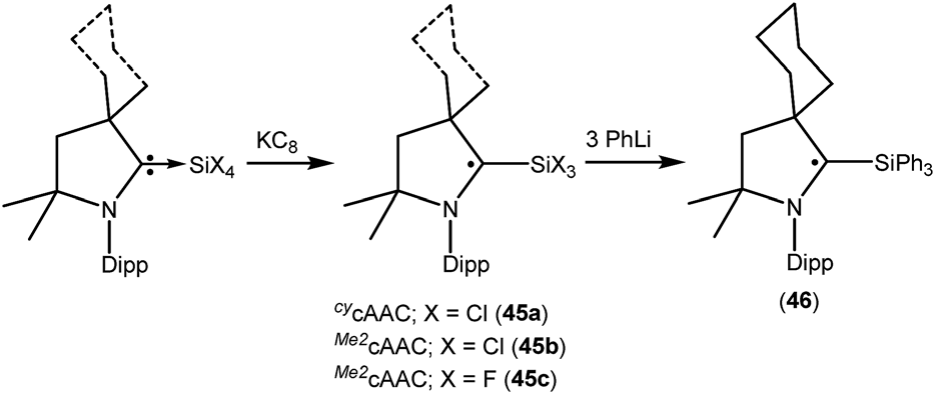
Synthesis of (cAAC˙)SiR_3_ (R = Cl, F or Ph).

We have also reported that the (cAAC)SiCl_4_ adduct can be converted to isolable trichlorosilylcarbene radicals (^*Cy*^cAAC˙)SiCl_3_ (**45a**) and (^*Me2*^cAAC˙)SiCl_3_ (**45b**) (Fig. 12) through a one equivalent KC_8_ reduction in hexane.^31^ The use of non-polar solvent (*n*-hexane) is important for the selective reduction of (cAAC)SiCl_4_ to (cAAC˙)SiCl_3_ (**45a–b**) at room temperature. Theoretical calculations show that the unpaired electron is mainly located on the carbene carbon of cAAC (52%), with a smaller contribution (23%) from the nitrogen atom of cAAC and the remaining 25% electron density is distributed over the Dipp/cAAC units and one of the Cl atoms. The EPR spectra of **45a–b** in C_6_D_6_ solution show multiple hyperfine lines. The EPR spectrum of **45a** reveals hyperfine coupling [*a*(^14^N) = 6.4 and 3.4 G (1 Cl) and 2.7 G (2 Cl), with three Cl atoms (I = 3/2, nat. abundance of ^35^Cl: 75.77%, ^37^Cl: 24.23%; gyromagnetic ratio = 1.20)] which suggests a partially hindered rotation of the SiCl_3_ group around the carbon–silicon bond. Very recently we have also reported the synthesis of a silicon trifluoride monoradical (^*Me2*^cAAC˙)SiF_3_ (**45c**; Fig. 12) from (^*Me2*^cAAC)SiF_4_ adduct by adopting a similar synthetic strategy as used for preparing (cAAC˙)SiCl_3_ (**45a–b**).^30^,^31^ The EPR spectrum of **45c** in hexane at room temperature shows multiple hyperfine coupling constants [*a*(^14^N) = 6.9 G, *a*(^29^Si) = 10 G, *a*(^19^F, 3F) = 16.8 G, *a*(^1^H, 3H) = 1.2 G.]. The trichlorosilylcarbene radical (**45a**) was directly converted to (^*cy*^cAAC˙)SiPh_3_ (**46**; Fig. 12) in 90% yield by substitution of the three chlorine atoms with phenyl groups using PhLi without affecting the radical center adjacent to the silicon atom.^32^ The X-band EPR spectrum of **46** exhibits three hyperfine lines due to coupling with one nitrogen nucleus (*a*(^14^N) = 5.4 G). Additionally, satellites for the silicon and three carbon atoms (*a*(^29^Si) = 8.0 G and *a*(^13^C) = 25 G), are also identified through simulation of the EPR spectrum of **46**.

**Fig. 13:**
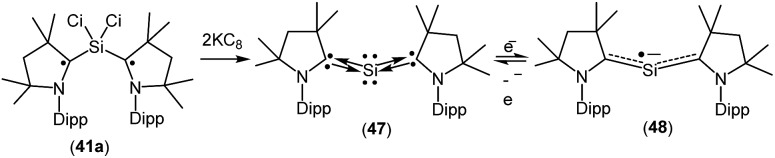
cAAC-stabilized siladicarbene and *in situ* generation of (^*Me2*^cAAC)_2_Si˙^–^.

A structurally characterized, stable siladicarbene (^*Me2*^cAAC)_2_Si (**47**, Fig. 13) was reported by us by the reduction of **41a** with two equivalents of KC_8_.^33^ Compound **47** has singlet spin ground state which has been confirmed by magnetic susceptibility and EPR measurements. The ^29^Si NMR spectrum of (^*Me2*^cAAC)_2_Si (**47**) exhibits a singlet at 66.71 ppm which is downfield shifted when compared with that of the precursor (^*Me2*^cAAC˙)_2_SiCl_2_ (**41a**) (4.13 ppm). However, the dark blue color of **47** suggests a small HOMO–LUMO gap. The experimental charge density calculations have confirmed the presence of two pairs of electrons on the silicon atom of **47**.^34^ Calculations at various levels of theory using the M05-2X/SVP optimized geometries predict that the triplet form is between 17.2 kcal mol^–1^ (M05-2X/TZVPP) and 18.5 kcal mol^–1^ (B3LYP/TZVPP//M05-2X/SVP) higher in energy than the singlet. CASSCF(2,2)/SVP calculations using the M05-2X/SVP optimized geometry gave coefficients of 0.96 for the closed-shell 2,0 configuration, –0.28 for the 1,1 configuration, and 0.0 for the 0,2 configuration which indicate that **47** has a closed-shell singlet with a non-negligible contribution from the singly excited state.

When **47** was stirred in THF at 298 K in the presence of potassium metal for 30 min a radical anion [(^*Me2*^cAAC)_2_Si˙^–^ (**48**); Fig. 13] was generated.^35^ The EPR spectrum of (^*Me2*^cAAC)_2_Si˙^–^ (**48**) reveals five hyperfine lines at *g* = 2.0058 indicating the coupling of a radical electron with two equivalent nitrogen nuclei (*a*(^14^N) = 5.89 G). Two satellites (*a*(^13^C) = 40 G; *I* = 1/2) are observed due to coupling with C_cAAC_ atoms which indicates that the unpaired electron is delocalized in the C–Si–C back bone of **48**. The Mulliken spin density plot of the radical anion (**48**) shows that the unpaired electron is delocalized between the two carbene carbon atoms of cAAC through the vacant d-orbitals of the silicon atom.

**Fig. 14:**
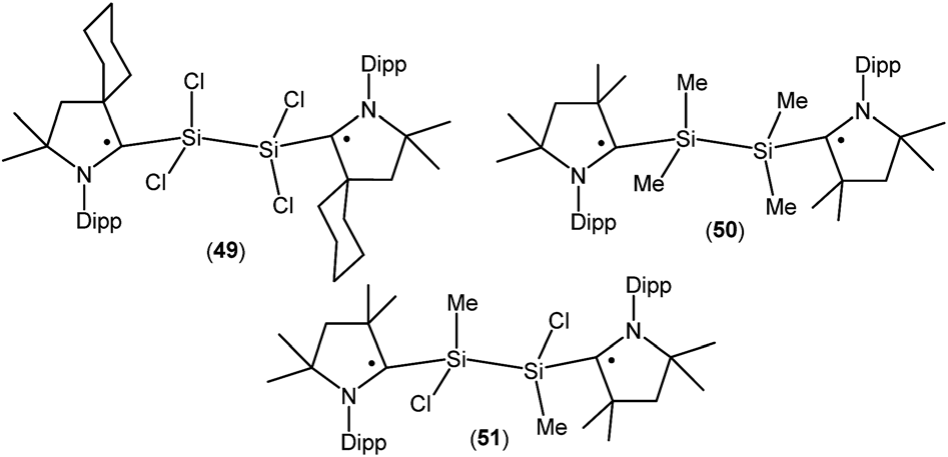
Silane-bridged dis- and biradicals.

Treatment of **45a** with one equivalent of KC_8_ in THF at low temperature generates a green- colored silane-bridged biradical disilicontetrachloride (^*cy*^cAAC˙)_2_Si_2_Cl_4_ (**49**, Fig. 14).^36^ Compound **49** can also be obtained from the direct reduction of (^*cy*^cAAC)SiCl_4_ with two equivalents of KC_8_. Theoretical calculations (M06-2X/TZVP//M06-2X/SVP level) revealed that **49** possesses an open shell singlet ground state with an unpaired electron residing on each C_cAAC_ atom having opposite spins. The open shell singlet state of **49** was found to be lower in energy than the triplet state by 2.8 kcal mol^–1^. Accordingly, **49** is EPR silent and reveals a chemical shift at 3.3 ppm in its ^29^Si{^1^H} NMR spectrum. Very recently the analogous compounds (^*Me2*^cAAC˙)SiMe_2_–SiMe_2_(^*Me2*^cAAC˙) (**50**; Fig. 14) and (^*Me2*^cAAC˙)SiMeCl–SiMeCl(^*Me2*^cAAC˙) (**51**; Fig. 14) were also reported by us.^29^ Compounds **50** and **51** were synthesized by a direct reduction of the commodity precursors Me_2_SiCl_2_ and MeSiCl_3_, respectively with KC_8_ in 1 : 2 molar ratios in the presence of one equivalent of cAAC. It has been observed that the replacement of chlorine atoms of **49** by methyl groups leads to the disbiradical **50**. The EPR spectrum of **50** at room temperature in hexane shows a 1 : 1 : 1 triplet with weak satellite signals because of hyperfine coupling with one nitrogen atom (*a*(^14^N) = 4.8 G) and with a silicon atom (*a*(^29^Si) = 25.7 G). No half-field signal was observed for **50**. Compound **51**, where one methyl and one chlorine atom is attached to each silicon center also shows paramagnetic behavior. EPR spectrum of **51** in hexane at room temperature shows hyperfine splitting with *a*(^14^N) = 4.0 G, *a*(CH_3_) = 5.3 G, *a*(^35,37^Cl) = 4.5 G, and *a*(^29^Si) = 16 G.^29^ Similar to compound **50**, EPR data of **51** indicates that there is no significant spin–spin interaction. Theoretical calculation (M06-2X/def2-TZVPP//M06-2X/def2-SVP) of the two molecules in the electronic OSS and triplet states suggest that the triplet states of **50** (–11.3 kcal mol^–1^), and **51** (–12.1 kcal mol^–1^) are lower in energy than the respective OSS states. The calculated spin density distributions show that the unpaired electrons are located at the carbene carbon and nitrogen atoms of the cAAC ligands for compounds **50** and **51**.

**Fig. 15:**
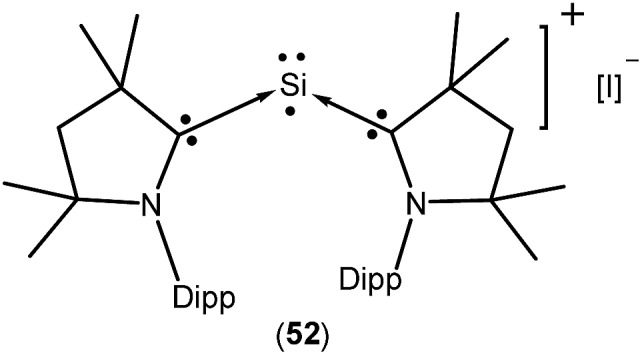
cAAC-stabilized monoradical cation of Si(i).

Isolation of a purple colored cationic silicon(i) radical [(^*Me2*^cAAC)_2_ Si:˙]^+^I^–^ (**52**; Fig. 15) was reported by So *et al.* in 2017 from the direct reaction of cAAC with H_2_SiI_2_ in THF or 1,2-dimethoxyethane solvent.^37^ Theoretical calculations showed that the unpaired electron is mainly localized on the Si atom (0.52 e) with a slight delocalization on one of the cAAC ligands. Room temperature EPR analysis in 1 : 1 toluene/THF solvent showed a three line hyperfine multiplet pattern (*a*(^14^N) = 18.5 MHz). This characteristic splitting pattern indicates that the radical is delocalized on only one of the cAAC ligands, which is consistent with the calculated spin densities of **52**. A pair of weak satellite signals (*a*(^29^Si) = 212 MHz) was observed in the EPR spectrum recorded at 115 K.

## Group 15 radicals

Phosphorus-centered radicals are attracting much attention as ligands for their use in spin-labelling experiments because of their orientation dependence of the large hyperfine coupling with ^31^P. The large hyperfine coupling constant provides details regarding much faster molecular movements than is possible with the widely used nitroxide probes. A number of cAAC-stabilized group-15 elements-centered radicals have been isolated by various groups which are given in Fig. 16.26a,^38^ Table 3 summarizes the spin densities of group 15 radical species.

**Fig. 16:**
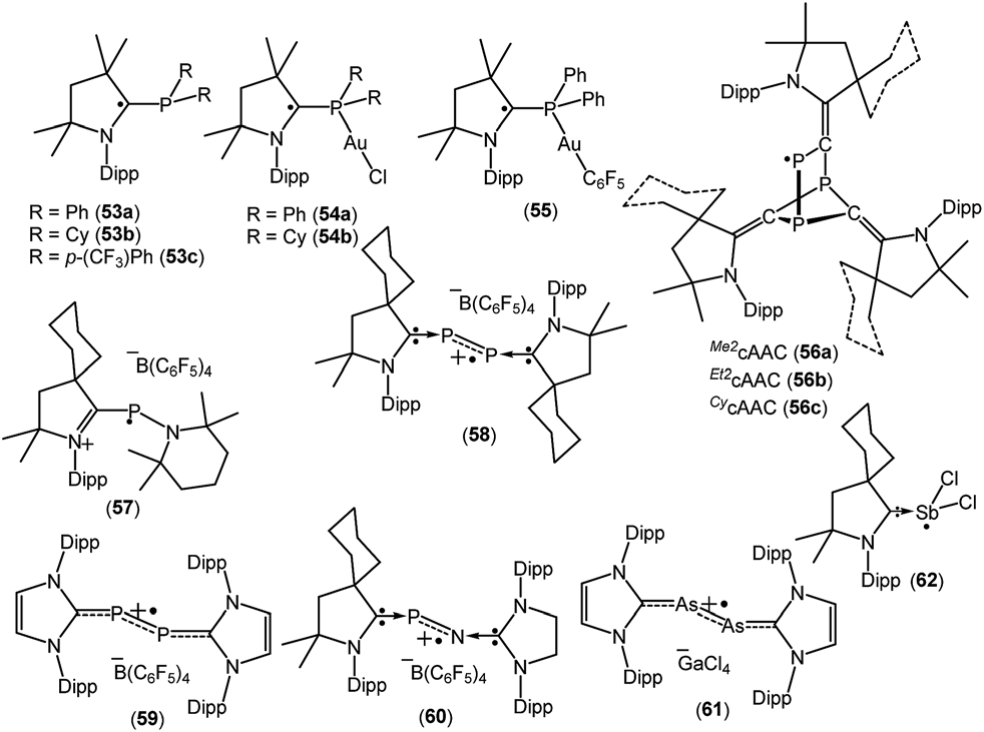
cAAC stabilized group-15 radicals.

**Table 3:** Type of radicals and spin densities of groups 15–16 species

Compounds	Spin density	Type of radical	Ref.
**53a–c**	(65–70%) C_carbene_, (20–25%) N_carbene_, 1% P	Monoradical	38*a*
**54a–b**, **55**	(60–67%) C_cAAC_, (23–28%) N_cAAC_, (1–2%) P, (0.1–0.5%) Au	Monoradical	38*a*
**56a–c**	73–76% P	Monoradical	38*b*
**57**	67% P, 16% N, 10% N_carbene_	Monoradical cation	38*c*
**58**	27% P (each), 14% N_cAAC_ (each)	Monoradical cation	38*d*
**59**	33% P, 44% P	Monoradical cation	38*d*
**60**	40% P, 18% N, 19% N_cAAC_	Monoradical cation	38*e*
**61**	41% As (each)	Monoradical cation	38*f*
**62**	90.7% Sb, 4.6% Cl, 3.9% Cl	Monoradical	38*g*
**63a–b**	75% C_cAAC_, 19% N_cAAC_, 3.6% Si, 5% P	Monoradical	39
**64**	81% C_cAAC_, 9% N_cAAC_, 6% Si (total), 6% P (total)	Monoradical anion	40
**65**	52% CO (40% O and 12% C), 26% N_carbene_ (total), 19% N_carbene_ (total)	Monoradical cation	41

Recently Alcarazo and co-workers have reported the successful isolation of cAAC-stabilized neutral α-radical phosphines (**53a–c**; Fig. 16), which were obtained by a one-electron reduction by KC_8_ from their corresponding cationic precursors.38*a* These compounds were characterized by X-ray diffraction and EPR spectroscopy. The hyperfine splitting in the EPR spectrum of **53a–c** differ and show distinct delocalization of spin density onto the adjacent nitrogen and phosphorus atoms. The extent of delocalization depends on the substituent at the phosphorus centre. Six signals were observed in the EPR spectra of **53a** and **53c**, which is due to the coupling of the electron spin with the ^31^P and ^14^N nuclei [*a*(^31^P) = 18.0 G and *a*(^14^N) = 6.0 G for **53a**, and *a*(^31^P) = 14.7 G and *a*(^14^N) = 6.1 G for **53c**]. In comparison to **53a** and **53c**, a triplet was observed for **53b** which exhibits almost negligible coupling with ^31^P nuclei (*a*(^31^P) = 1.0 G), but similar coupling with ^14^N (*a*(^14^N) = 5.17 G) as in **53a** and **53c**. Theoretical calculations revealed that the spin density in **53a–c** is mainly located at the cAAC ligands (*ca.* 65–70% on the carbene carbon atoms, *ca.* 20–25% on the N atoms), but only some residual spin density at the P center (*ca.* 1%). Interestingly, these α-radical phosphines (**53a–b**) can form Au(i) complexes [{(^*Me2*^cAAC)PPh_2_}-AuCl]˙ (**54a**), [{(^*Me2*^cAAC)PCy_2_}-AuCl]˙ (**54b**) and [{(^*Me2*^cAAC)PPh_2_}AuC_6_F_5_]˙ (**55**) without affecting the radical centre, thus serving as spin-labelled ligands.38*a* The EPR spectra of **54–55** is entirely dominated by hyperfine coupling with ^31^P and ^14^N nuclei and the absence of hyperfine coupling with ^197^Au (*I* = 3/2, natural abundance 100%) indicates that the metal and the radical system are electronically isolated from each other. Theoretical calculations confirmed that the unpaired electron in **54a–b** and **55** is primarily located on the carbene carbon atom (*ca.* 60–67%), with significant contributions from the nitrogen atom of cAAC (23–28%), and rather small contributions from phosphorus (*ca.* 1–2%) and the predicted spin densities at gold centre are only residual (0.1–0.5%).38*a*

In 2018, Grützmacher *et al.* reported a series of tricarbontriphosphide tricyclic radicals [(^*Me2*^cAAC)_3_C_3_P_3_ (**56a**); (^*Et2*^cAAC)_3_C_3_P_3_ (**56b**); (^*Cy*^cAAC)_3_C_3_P_3_ (**56c**)] (Fig. 16). These were synthesized from a one equivalent reduction of the corresponding precursors cAAC=C=P–[P(O){(N-Dipp)CH}_2_] with KC_8_.38*b* The reduction of cAAC=C=P–[P(O){(N-Dipp)CH}_2_] leads to the formation of the carbene-bound CP radical [cAAC=C=P]˙, which further trimerizes to give the tricyclic radical [(cAAC)_3_C_3_P_3_]˙ (**56a–c**). The EPR spectra of the hexane solutions of **56a–c** at room temperature show a doublet because of the large isotropic ^31^P hyperfine interaction (*a*_iso_ ≈ 187 MHz), which indicates that a large percentage of the spin density is localized on one of the phosphorus nuclei. Small ^14^N hyperfine couplings [*a*(^14^N) = 11 MHz for **56a**; *a*(^14^N) = 12 for **56b** and *a*(^14^N) = 9 MHz for **56c**] were observed. These estimations of the spin populations from EPR (0.73–0.74 e on P) agreed well with DFT results (0.76 e on P).38*b* In 2010, Bertrand reported that ^*Cy*^cAAC reacts with R_2_NPCl_2_, [(2,2,6,6-tetramethylpiperidino)phosphine dichloride] to generate [(^*Cy*^cAAC)P(Cl)(NR_2_)]^+^Cl^–^ which can be reduced to aminophosphinidene [(^*Cy*^cAAC)PNR_2_] with magnesium metal.38*c* This aminophosphinidene was converted to its stable radical cation [(^*Cy*^cAAC)(NR_2_)P]˙^+^(C_6_F_5_)_4_B^–^ (**57**) by a one-electron oxidation with Ph_3_C^+^B(C_6_F_5_)_4_^–^ in benzene at room temperature (Fig. 16). The radical cation **57**^+^˙ was characterized by single crystal X-ray diffraction and by EPR spectroscopy.38*c* The EPR spectrum of **57**^+^˙, in fluorobenzene, at room temperature, shows a doublet of multiplets (*g* = 2.007) due to a large hyperfine coupling with ^31^P [*a*(^31^P) = 99 G] and a small coupling with one or two ^14^N nitrogen nuclei [*a*(^14^N) ≈ 4 G]. Consistent with the EPR data, theoretical calculations confirmed that the spin density in **57**^+^˙ is localized mainly at phosphorus (67%) with small contributions from the nitrogen atoms (16% for piperidino nitrogen and 10% for cAAC nitogen).38*c* The authors have mentioned that the exceptional stability of **57**^+^˙ is partly due to steric factors but more importantly because of cationic substituent which prevents the dimerization by electrostatic repulsion.

The Bertrand group has shown that white phosphorus (P_4_) reacts with ^*Cy*^cAAC to give 2,3-diphosphabutadiene [(^*Cy*^cAAC)_2_P_2_] which can be considered as a diatomic phosphorus molecule stabilized by two cAAC substituents.38*d* The cyclic voltammogram of [(^*Cy*^cAAC)_2_P_2_] in THF solution, containing 0.1 M *n*-Bu_4_NPF_6_ as electrolyte, shows a reversible one-electron oxidation at *E*_1/2_ = –0.536 V *versus* Fc^+^/Fc, which indicates the formation of the radical cation. The chemical synthesis of the cationic radical [(^*Cy*^cAAC)_2_P_2_]˙^+^(C_6_F_5_)_4_B^–^ (**58**, Fig. 16) was quantitatively achieved from the reaction of [(^*Cy*^cAAC)_2_P_2_] and [Ph_3_C][B(C_6_F_5_)_4_] in an equimolar ratio in toluene at room temperature.38*d* The cationic radical **58**^+^˙ was characterized by single crystal X-ray diffraction and EPR measurements. The EPR spectrum of a fluorobenzene solution of **58**^+^˙ at room temperature shows a triplet of quintets due to a large coupling with two equivalent phosphorus nuclei (*a*(^31^P) = 42 G) and a small coupling with two nitrogen nuclei (*a*(^14^N) = 3 G). Theoretical calculations indicate that the spin density in **58**^+^˙ is distributed between the two phosphorus atoms (0.27 e at each P) and the two nitrogen atoms of the cAAC ligands (0.14 e at each N). It is important to mention that the unpaired electron in a similar radical cation [(NHC)_2_P_2_]˙^+^(C_6_F_5_)_4_B^–^ (**59**, Fig. 16) is nearly exclusively localized at phosphorus atoms (0.33 e and 0.44 e), with a contribution of less than 0.07 e for any other atoms.38*d* Phosphorus mononitride (PN) has attracted huge interest because of its existence in the interstellar medium and the atmospheres of Jupiter and Saturn. In 2010, Bertrand and co-workers successfully isolated the phosphorus mononitride (NHC′)NP(^*Cy*^cAAC) in molecular form which was stabilized by two carbene units.38*e* The precursor, (NHC′)NH [NHC′ = {CH_2_N(dipp)}_2_C:] was synthesized by treatment of NHC with bromine, followed by addition of aqueous ammonia (NH_4_OH). The (NHC′)NH was then deprotonated with *n*-BuLi and the *in situ* generated anion (NHC′)N^–^ was treated with PCl_3_ to obtain (NHC′)NPCl_2_, which was reduced with magnesium in the presence of one equivalent of cAAC to obtain (NHC′)NP(^*Cy*^cAAC) in good yield.38*e* The cyclic voltammetry of (NHC′)NP(^*Cy*^cAAC) in a THF solution containing 0.1 M *n*-BuNPF_6_ as electrolyte shows a reversible one-electron oxidation at *E*_1/2_ = –0.51 V *versus* Fc^+^/Fc, indicating the formation of the corresponding radical cation [(NHC′)NP(^*Cy*^cAAC)]^+^˙ (**60**^+^˙, Fig. 16). The chemical synthesis of the **60**^+^˙ was achieved from the 1 : 1 reaction of (NHC′)NP(^*Cy*^cAAC) with [Ph_3_C][B(C_6_F_5_)_4_] in toluene at room temperature. The compound [(NHC′)NP(^*Cy*^cAAC)]^+^˙(C_6_F_5_)_4_B^–^ (**60**) was characterized by a single crystal X-ray diffraction and EPR spectroscopy.38*e* The EPR spectrum of a fluorobenzene solution of **60**^+^˙ at room temperature displays a doublet due to a large coupling with phosphorus [*g* = 2.0048; *a*(^31^P) = 44 G] while no coupling was observed with the nitrogen atom. This observation indicates that the major spin density is localized on the phosphorus atom. Theoretical calculations indicated that the spin density in **60**^+^˙ is mainly distributed on the phosphorus atom (0.40 e) along with the central nitrogen atom (0.18 e) and the nitrogen atom of the cAAC ligand (0.19 e).38*e* Robinson *et al.* in 2013 reported that the oxidation of a carbene-stabilized diarsenic compound, L:As–As:L [L: = :C{N(2,6-^i^Pr_2_C_6_H_3_)CH}_2_], with gallium chloride in a 1 : 2 ratio in Et_2_O afforded the first arsenic radical [L:AsAs:L]˙^+^[GaCl_4_]^–^ (**61**, Fig. 16) to be structurally characterized in the solid state.38*f* The room-temperature EPR spectrum of **61**˙^+^ in fluorobenzene displayed a broadened septet (*g* ≈ 2.05) resulting from a large hyperfine coupling with two equivalent ^75^As (*I* = 3/2) nuclei.38*f* Bertrand *et al.* in 2014 reported the first molecular example of a neutral antimony centered radical [(^*Cy*^cAAC)SbCl_2_]˙ (**62**; Fig. 16) from the one equivalent reduction of (cAAC)SbCl_3_ adduct by KC_8_.38*g* The radical **62** has been characterized by EPR spectroscopy as well as by single-crystal X-ray diffraction. X-band EPR spectrum of **62** in benzene solution at room temperature shows a septet with significant isotropic hyperfine constants due to the coupling with two nearly equivalent chlorine nuclei (*S* = 3/2). The simulation of the EPR spectrum of **62** shows various hyperfine couplings [*a*(^121^Sb) = 0.003 (natural abundance 57%), *a*(^123^Sb) = 0.003 (natural abundance 43%), and *a*(^35^Cl) = 4.472, *a*(^35^Cl) = 4.472 G and calculated values: *a*(^121^Sb) = 0.004, *a*(^123^Sb) = 0.004, *a*(^35^Cl) = 3.217, *a*(^35^Cl) = 3.533 G]. The experimental EPR investigations support the theoretical findings, which indicate that the spin density is mostly located on antimony (90.7%) with minor contributions from the two chlorine atoms (4.6% and 3.9%).38*g*

**Fig. 17:**
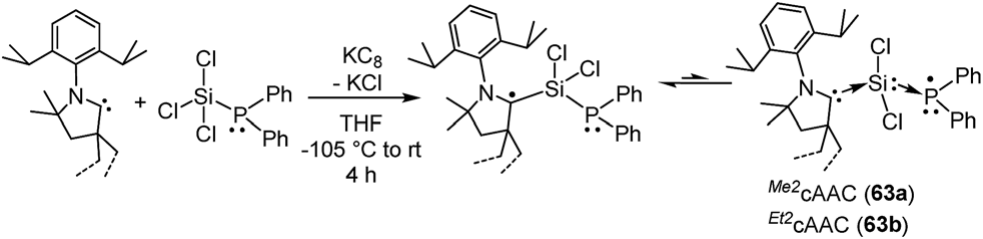
Radicals of silicon and phosphorus stabilized by cAAC.

In this regard, we were interested to isolate radicals of mixed group-14 and group-15 elements. Accordingly, we reported two stable radicals [(^*Me2*^cAAC˙)Si(Cl_2_)(PPh_2_) (**63a**) and (^*Et2*^cAAC˙)Si(Cl_2_)(PPh_2_) (**63b**); Fig. 17] from the commonly used precursors trichlorosilane and diphenylchlorophosphine.^39^ Compounds **63a** and **63b** were isolated from the direct reduction of Ph_2_PSiCl_3_ by one equivalent of KC_8_ in the presence of one equivalent of cAAC; all the reactions were initiated at low temperature (∼–105 °C) in THF. Both the compounds were structurally characterized. The calculated Mulliken spin density plots of **63a** and **63b** suggest that the unpaired electron is mostly located on the carbene carbon (75–76%), with a comparatively lower contribution from the nitrogen atom (18–20%) of the cAAC. Moreover, some finite occupancy over the phosphorus (∼3%) and one of the chlorine atoms (1%) was also observed. The EPR spectrum of **63a** in toluene could be simulated [*a*(^31^P) = 15.6 G (calc. 20.5 G), *a*(^14^N) = 6.5 G (calc. 4.2 G), *a*(^35^Cl) = 4.1 G (calc. 3.1 G)]. A ^29^Si satellite coupling (4.7% nat. abundance, *I* = 1/2) could be observed for **63b** [*a*(^29^Si) = 10 G (calcd 13.4 G); 3.6% spin density]. It is worth to note that **63b **contains an apparently mobile ethyl substituent of low symmetry which leads to a strong temperature-dependent EPR spectrum between 183 and 340 K. Thus, simulation could not be achieved within the accessible temperature range for **63b**. The redox property of **63a** was also investigated by cyclic voltammetry measurements in THF solution which shows a one electron quasi-reversible process at *E*_1/2_ = –0.86 V against Cp*_2_Fe/Cp*_2_Fe^+^, suggesting the formation of the anion [(^*Me2*^cAAC·)Si(Cl_2_)(PPh_2_)]^–^.

**Fig. 18:**
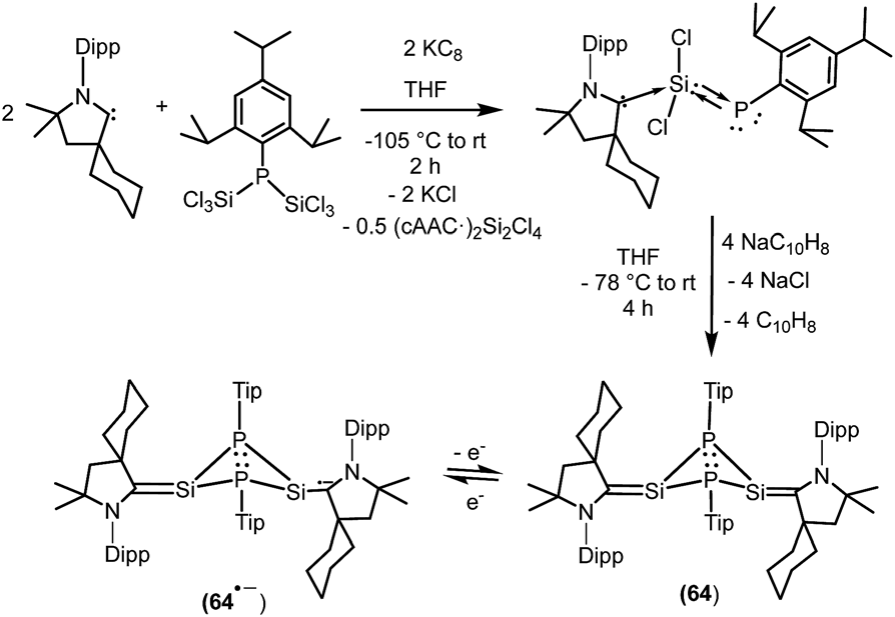
Radical compounds of silicon and phosphorus stabilized by cAAC.

**Fig. 19:**
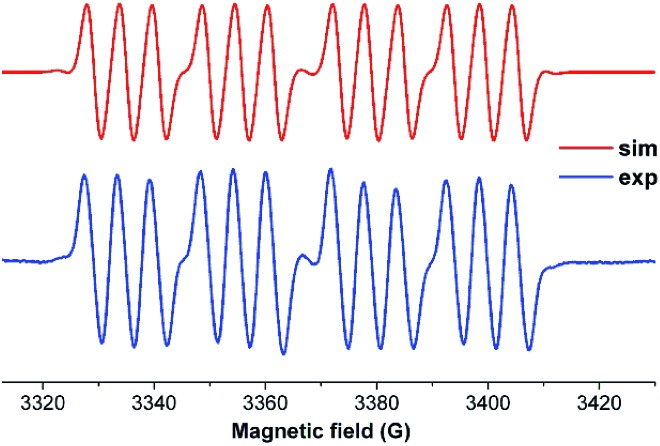
EPR spectrum of radical anion [(^*Cy*^cAAC)Si(P-Tip)]_2_˙^–^ (**64˙^–^**) in toluene at 285 K.

We have also prepared, in a step-wise synthesis, a dimeric heavier analogue of ketenimine with phosphorus and silicon atoms [(^*Cy*^cAAC)Si(P-Tip)]_2_ (**64**) (Fig. 18).^40^ The cyclic voltamogram of **64** revealed a one-electron quasi-reversible process (*E*_1/2_ = –0.87 V against Cp*_2_Fe/Cp*_2_Fe^+^) suggesting the formation of the radical anion [(^*Cy*^cAAC)Si(P-Tip)]_2_˙^–^ (**64˙^–^**) which could not be isolated but could be detected by EPR spectroscopy. Thus, the X-band EPR spectrum of the *in situ* generated radical anion (**64˙^–^**) at 285 K in toluene solution shows twelve well-resolved lines of equal intensity (Fig. 19). The splitting pattern shows a doublet of doublets, where each component splits further into three equidistant lines. The latter splitting is assigned to the coupling with one ^14^N nucleus (*a*(^14^N) = 5.9; *I* = 1) which is in the typical range for cAAC centered radicals. The two larger doublet hyperfine splittings [*a*(^31^P) = 44.1 G and *a*(^31^P) = 20.6 G] are due to coupling with two inequivalent ^31^P nuclei (*I* = 1/2). A satellite for the ^29^Si nuclei (*a*(^29^Si) = 11 G) coupling was also observed in the EPR spectrum of **64˙^–^**. Theoretical calculations and simulation of EPR spectrum of **64˙^–^** reveal the non-equivalence of the two phosphorus nuclei and also the presence of the unpaired electron on one of the two carbene carbon atoms.^40^

## Group 16 radicals

**Fig. 20:**
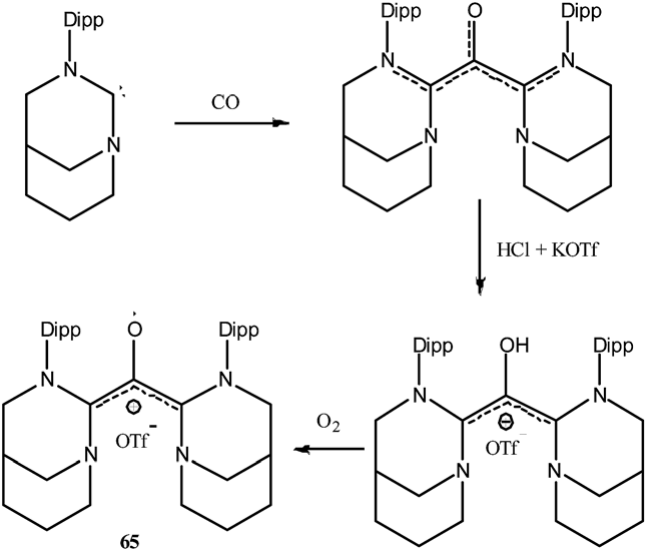
Synthesis of oxyallyl radical cation **65**˙^+^.

To the best of our knowledge there is no group 16 radical reported in the literature which is stabilized by cAAC. This could be because chalcogens (E = O, S, Se, Te) form strong adducts with carbene and the interaction can be better represented as chalcogen–carbene double bond (carbene = E). As carbene = E is electronically satisfied and has no unpaired electron, chalcogens do not form radical species in presence of carbenes. Chemistry of carbenes with substituted chalcogens (R-E) is not well established and no radical species have been isolated so far. However there is only one report where an air stable oxyallyl radical cation **65**˙^+^ has been isolated by Bertrand and co-workers using a NHC based ligand system (Fig. 20).^41^

## Conclusions and perspectives

The area of main-group element radicals is expanding very rapidly with isolation of many exotic and hitherto inaccessible species. It has been observed that all these systems, radicals, radical ions, biradicals, biradicaloids and disbiradicals are stabilized primarily by the steric bulk of the ligands to prevent their dimerization or polymerization. However, recent examples indicate that the radical intermediates can also be stabilized by carbene ligands. NHCs stabilize the radical intermediates mainly by σ-donation whereas cAACs switch their bonding nature depending upon the accumulation of electron density around the radical center because of their additional π-accepting property. The donor and acceptor behavior of the cAACs make it possible to enable the isolation of new types of radicals which were not achievable by conventional methods or by the use of NHC. Thus, the silylene radical anion could be characterized only at low temperatures while the cAAC coordinated silylene radical anion is stable at room temperature. Similarly, dichlorosilylene stabilized by NHC [(NHC)SiCl_2_] exists in its non-radical monomeric form while SiCl_2_ stabilized by two cAACs [(cAAC˙)_2_SiCl_2_] behaves as biradical. We have reported in this review the successful efforts in the isolation of main-group radical compounds containing carbon, aluminum, silicon and phosphorus stabilized by cAACs. These efforts are the result of development of new synthetic methods involving, first the formation of cAAC main-group adducts, followed by reduction of the adducts by a variety of reducing agents. The role of the cAAC ligand in stabilizing the radical species has been delineated by theoretical methods which throws light on the unique electronic features of this ligand. These results on main-group radical compounds will, we are sure, trigger enormous interest in the coming years not only in the isolation of newer systems but also in finding applications for these new families of stable main-group radicals.

## Conflicts of interest

There are no conflicts to declare.
